# The Effect of Ultrasonication on the Fibrillar/ Oligomeric Structures of Aβ_1−42_ at Different Concentrations

**DOI:** 10.1007/s10930-023-10138-0

**Published:** 2023-08-27

**Authors:** Nassim Faridi, Maryam Sanjari-Pour, Ping Wang, S. Zahra Bathaie

**Affiliations:** 1 Department of Clinical Biochemistry, Faculty of Medical Sciences, Tarbiat Modares University, P.O. Box. 14115-133, Tehran, Iran; 2 Institute for Natural Products and Medicinal Plants, Tarbiat Modares University, Tehran, Iran; 3 College of Pharmaceutical Sciences, Zhejiang University of Technology, Hangzhou, 310014 People’s Republic of China; 4 UCLA-DOE Institute, University of California, Los Angeles, CA USA

**Keywords:** Amyloid Fibrils, Protein Aggregation, Amyloid β Oligomers, Ultrasonic Pulses, Cell Toxicity

## Abstract

The number of disease states linked the aberrant regular protein conformations to oligomers and amyloid fibrils. Amyloid beta 1–42 (Aβ_1−42_) peptide is very hydrophobic and quickly forms the β-rich structure and fibrillar protein aggregates in some solutions and buffer conditions. Ultrasonication pulses can disrupt amyloid fibrils to smaller fragments and produce Aβ_1−42_ peptides of different sizes and oligomers. Herein, we investigated the effects of buffer and ultrasonication on Aβ_1−42_ structure at low and high concentrations. After ultrasonication, the Western blot results showed that Aβ_1−42_ fibrils were disaggregated into different sizes. The transmission electron microscopy results indicated Aβ_1−42_ at low concentration (25 µM) in Ham’s/F12 phenol red-free culture medium formed short-size fragments and oligomers. In comparison, Aβ_1−42_ at higher concentration (100 µM) formed fibrils that break down into smaller fragments after ultrasonication. However, after regrowth, it formed mature fibrils again. Cell viability assay indicated that Aβ_1−42_ oligomers formed at a low concentration (25 µM) were more toxic to PC12 cells than other forms. In conclusion, by applying ultrasonication pulses and controlling peptide concentration and buffer condition, we can rich Aβ_1−42_ aggregates with a particular size and molecular structure.

## Introduction

Protein misfolding and aggregation have been introduced as the molecular mechanism of some neurodegenerative diseases. Aggregated conformers are classified based on their size, structure, and solubility. Recently, highly toxic Amyloid beta (Aβ) oligomers have been introduced as critical determinants in aggregate-induced toxicity [1–3]. Aβ is one of the most critical extracellular aggregates responsible for Alzheimer’s disease (AD). Therefore, Aβ peptides with different lengths, Aβ_1−40_ or Aβ_1−42_, have been used in different in vitro studies. They could exist as a monomer or larger soluble entities called Aβ oligomers with non-fibrillar structures, and eventually insoluble fibrils. The soluble oligomers of Aβ_1−42_ contain mixed intermolecular parallel and intramolecular antiparallel β-sheets. β-amyloid species have different types of assemblies: dimers, trimers, protofibrils, annular or pore-like oligomers, and spherical (globulomers) [4–6]. Aβ fibrils are unbranched, long supramolecular assemblies containing in-registered parallel β-sheets.

The cytotoxicity of Aβ_1−42_ is related to the soluble oligomers that, due to their interaction with the lipid bilayer of the cell membrane and pore formation, cause an uncontrolled ion flux [5, 7, 8]. It is because oligomeric Aβ_1–42_ can form the β-barrel in the cell membrane and permeabilize cells [9–11]. As mentioned above, different aggregated morphologies in neuronal cells exhibit different degrees of toxicity, and soluble oligomers are generally more toxic than amyloid fibrils [2, 12, 13]. Deposition of these aggregates is associated with various degenerative diseases, including AD, prion disease, and dialysis-related amyloidosis [14]. The in vitro protein aggregation mechanism is sensitive to subtle differences in environmental conditions such as buffer composition, agitation, and protein concentrations. Even tiny changes in protein/ peptide concentration can lead to the formation of specific types of oligomers and intermediates [15–17]. In vitro studies indicated that a low concentration of Aβ_1−42_ controls oligomers’ formation and favors a modest conformational conversion into fibrils. In contrast, its higher concentration would promote heterogeneous nucleation and aggregation to form fibrils of different sizes [18, 19].

Ultrasonication as an environmental factor has opposite effects on the formation and breakdown of fibrils [20]. Ultrasonic-dependent fragmentation is a fundamental approach to breaking Aβ_1−42_ fibrils into smaller aggregates [20, 21]. The main reason is the repeated growth and collapse of bubbles, which are under the control of negative or positive pressures [22–24].

Because of the tendency to aggregation and fibril formation of Aβ_1−42_ peptide in some conditions and the nature of insoluble particles formed, in the present study, we tried to find a method and situation to dissolve the insoluble aggregates and then reform the monomeric and oligomeric structures for in vitro studies. Thus, we decomposed Aβ_1−42_ large fibrils in phosphate-NaCl buffer using ultrasonication. After recovering the monomers, oligomeric structures formed through ultrasonication. For this purpose, we modeled low and high concentrations of Aβ_1−42_ peptide in different buffers using different techniques.

## Materials & Methods

### Materials

Aβ_1−42_ was bought from Sigma-Aldrich (St. Louis, USA) and GL Biochem. (Beijing, China) as the lyophilized powder. Hexafluoroisopropanol (HFIP) was purchased from Merck Co. (Darmstadt, Germany). Thioflavin T (ThT), thiazolyl blue tetrazolium bromide (MTT), 8-Anilinonaphthalene-1-sulfonate (ANS), and Hoechst were purchased from Sigma-Aldrich Chem. Co. (St. Louis, USA). The PC12 rat pheochromocytoma cell line was purchased from Pasture Institute (Tehran, Iran). Cell culture plates were acquired from SPL (Beijing, China). Primary antibodies (ab201060 and ab224275) and the secondary antibody (ab6721) were bought from Abcam (Abcam Inc., Cambridge, MA). ECL Plus Kit was purchased from Bio-Rad (Bio-Rad Laboratories Inc., Hercules, CA, USA). Alexa Fluor 594 (clone Poly4064) was purchased from BioLegend (San Diego, CA, USA). PVDF was purchased from GE Healthcare (Biosciences, Stockholm, Sweden). All other materials were of analytical grade.

### Methods

**Preparation of Aβ Conformers.** Aβ fibrils were formed according to the method described previously by us. Briefly, Aβ_1−42_ (220 µM) was dissolved in 10 mM Na_2_HPO_4_, 100 mM NaCl, pH 7.4, and incubated at 37 ºC up to 5 h [25].

Other Aβ_1−42_ peptide conformers were prepared by dissolving its powder in cold HFIP at a 2 mg/ml concentration and incubated at room temperature (RT) for 1 h. Then, the solution was divided into 25 µl aliquots, and HFIP was evaporated. The resulting Aβ_1−42_ films were stored at − 20 °C until further experiments. Before any experiment, the peptide was dissolved in DMSO at a final concentration of 5 mM. Then resuspended in the suitable buffer as follows and treated with ultrasonication.

The high fibril concentration was formed by resuspension of this peptide in 10 mM HCl at a final concentration of 100 µM and incubating at 37 °C for 24 h.

The lower fibril concentration was prepared by diluting this solution to 25 µM in Ham’s/F12 phenol red-free culture medium.

Finally, preparation and propagation of oligomers were done by resuspension of Aβ_1−42_ solution in DMSO into Ham’s/F12 (phenol red-free) at a final concentration of 100 µM incubated at 4 °C for 24 h [26].

**Ultrasonication Treatments.** Aβ_1−42_ fibrils were placed on a water bath-type Ultrasonicator ( EngoTech, Zurich, Switzerland). Ultrasonication pulses were applied to both low (25 µM) and high (100 µM) concentrations of Aβ_1−42_ solution for 120 min. The sonication output was set to 60 Hz and 280 watts, and the temperature was maintained at 4 °C throughout the treatment. The Aβ_1−42_ solution samples were named ultrasonicated high concentration (USH) and ultrasonicated low concentration (USL). In addition, a sample containing a high concentration of Aβ was also prepared and named as a non-ultrasonicated high concentration (NUSH).

**Transmission Electron Microscopy (TEM).** Amyloid conformers (NUSH, USH, and USL) were immediately placed on the 400-mesh carbon-coated copper grids. After 1 min incubation for adsorption of bio-macromolecules on the grid surface, the excess solution was removed with filter paper. Next, the grid’s surface was washed with deionized water. Then, the grids were negatively stained using 2% (w/v) uranyl acetate. After 15 min, the dried grids were used for TEM analysis. The electron micrographs were acquired using a transmission electron microscope (Model-EM208S) at 100 kV with a magnification of 200 K×. The images were prepared using a Digital camera. The electron micrographs were taken at Partow Rayan Rastak, Tehran, Iran.

**Western Blotting.** For Western blot, Aβ_1−42_ conformers were diluted to 2.5 mM in sample buffer. Then, the diluted samples were loaded onto the 12.5% SDS-PAGE. After electrophoretic separation, they were transferred to a polyvinylidene difluoride membrane (PVDF). The membranes were blocked with 3% BSA in Tris-buffered saline (TBS; 25 mM Tris–HCl, pH 7.4, 0.9% NaCl) containing 0.1% Tween 20 (TBS-T), washed for 10 min with TBS-T, and incubated with specific primary Aβ_1−42_ antibody (Ab11132) overnight at 4 °C. The day after, the membrane was washed three times, 10 min each, with TBS-T. Then, the membrane was incubated with horseradish peroxidase-conjugated anti-rabbit IgG Goat antibody in TBS-T containing 0.5% BSA for 1 h at RT. Next, the membrane was rewashed with TBS-T three times, 10 min each, and then with TBS. Immune-reactive bands were visualized by the enhanced chemiluminescence (ECL) method on X-ray films. Subsequently, intensities of bands on the SAS-PAGE and WB membrane were quantified by using *ImageJ* software version 1.49t (NIH approved). So, the bands with less than 10 kDa, in the range of 10–50 kDa, and more than 50 kDa were considered the monomer, oligomer and fibril, respectively.

**Dot Blot.** Each Aβ_1−42_ conformer was transferred and spotted (1 µg) on PVDF. Afterward, the membrane was blocked with 10% BSA in TBS-T for 1 h at 37 °C. Next, it was incubated with rabbit polyclonal anti-Aβ_1−42_ antibody for 2 h, at RT. Then the blots were washed in washing buffer, incubated with the appropriate horseradish peroxidase-conjugated secondary antibody at RT for 1 h, and developed with ECL.

**ThT Assay.** Aβ_1−42_ samples were diluted with ThT (0.4 µM ThT in 50 mM Na_2_HPO_4_ and 0.05% (w/v) NaN_3_, pH 7.4). The fluorescence intensity (FI) was measured using a Cytation 3 microplate reader (BioTek Instruments, Inc. Winooski, VT, USA). The FI of ultrasonicated and non-ultrasonicated Aβ_1−42_ at different time intervals of protein incubation at 37 °C was recorded at excitation and emission wavelengths of 450 and 482 nm, respectively.

**ANS Fluorescence Assay.** Diluted solutions (10 µM) of Aβ_1−42_ in all forms of NUSH, USH, and USL were exposed to 20 µM of ANS at different incubation time points. The ANS fluorescence intensity at 500 nm was read after the excitation of the samples at 380 nm at different time intervals. The data was obtained using a Cytation 3 microplate reader.

**Circular Dichroism (CD).** Far-UV (190–260 nm) CD spectra explored changes in the secondary structure of the diluted conformers of oligomers, NUSH, USH, and USL solutions of Aβ_1−42_ in 10 mM PBS, pH 7.4. These conformers were evaluated using a JASCO-J-810 Spectropolarimeter (JASCO Corporation, Tokyo, Japan), using a 5-mm path length cuvette at 25 °C. Spectra were recorded with wavelength intervals of 1 nm, a response time of 4 s, and a scan rate of 100 nm/min. Each spectrum is the average of 4 scans of samples after subtraction of the related baseline. The noise component in data was smoothed using the JASCO J-810 software, including the fast Fourier-transform noise reduction method, which allows enhancement of most noisy spectra without distorting their peak shapes. The amounts of the secondary structures of the peptides were estimated using the Protein Secondary Structure Estimation Program (JWSSE-480), J-800 for windows. Yong plots were used as references for α-helix, β-sheet, β-turn, and random coil.

**Cell Toxicity Assay.** Aβ_1−42_ toxicity was evaluated by cell viability assay using MTT. Therefore, we firstly grew rat adrenal pheochromocytoma cells (PC12) in RPMI-1640 medium supplemented with 5% (v/v) fetal bovine serum (FBS), 10% (v/v) horse serum, and 1% (v/v) penicillin/ streptomycin. Then, PC12 cells were trypsinized and, after washing, were seeded in 96-well plates. After 24 h, different types of Aβ_1−42_ peptides (NUSH, USH, and USL) were separately added to the PC12 cells. The cells were incubated for an additional 24 h at 37 °C, and then the MTT solution (20 µl of 5 mg/ml) was added to the wells. After 4 h of incubation with MTT, the media were removed and replaced with 200 µl of DMSO. The color intensity of the formazan solution was quantified using an ELISA plate reader (Tecan, Zurich, Switzerland) at 570 nm. The control (untreated) cells were assumed 100% viable, and the viability of the Aβ_1−42_ treated cells was calculated relative to the control and expressed as the percentages (%) of viability.

**Immunofluorescence Assay of Aβ**_**1− 42**_ **Cellular Uptake.** PC12 cells were grown in RPMI-1640 medium supplemented with 5% fetal bovine serum, 10% horse serum, and 1% penicillin/ streptomycin. Then, cells were exposed to the IC_50_ value of Aβ_1− 42_ conformers. After 24 h, treated cells were washed with PBS and fixed in 70% chilled methanol for 10 m at RT. After PBS washing, nuclei were permeabilized in 0.3% Triton-X for 10 m at RT. Next, PC12 Cells were washed with PBS, and unspecific binding sites were blocked with 1% BSA for 30 m at RT. Then, cells were subjected to primary Aβ_1− 42_ antibody at RT overnight. After that, cells were incubated with Alexa Fluor 594, as a fluorescent secondary antibody, for 1 h at RT, followed by counterstaining with Hoechst 33,258 solution. Finally, cells were scanned and imaged with a Cytation™ 3 microscope (BioTek Instruments, Inc. Winooski, VT, USA).

## Results

### The TEM Analyzes

**Fig. 1:**
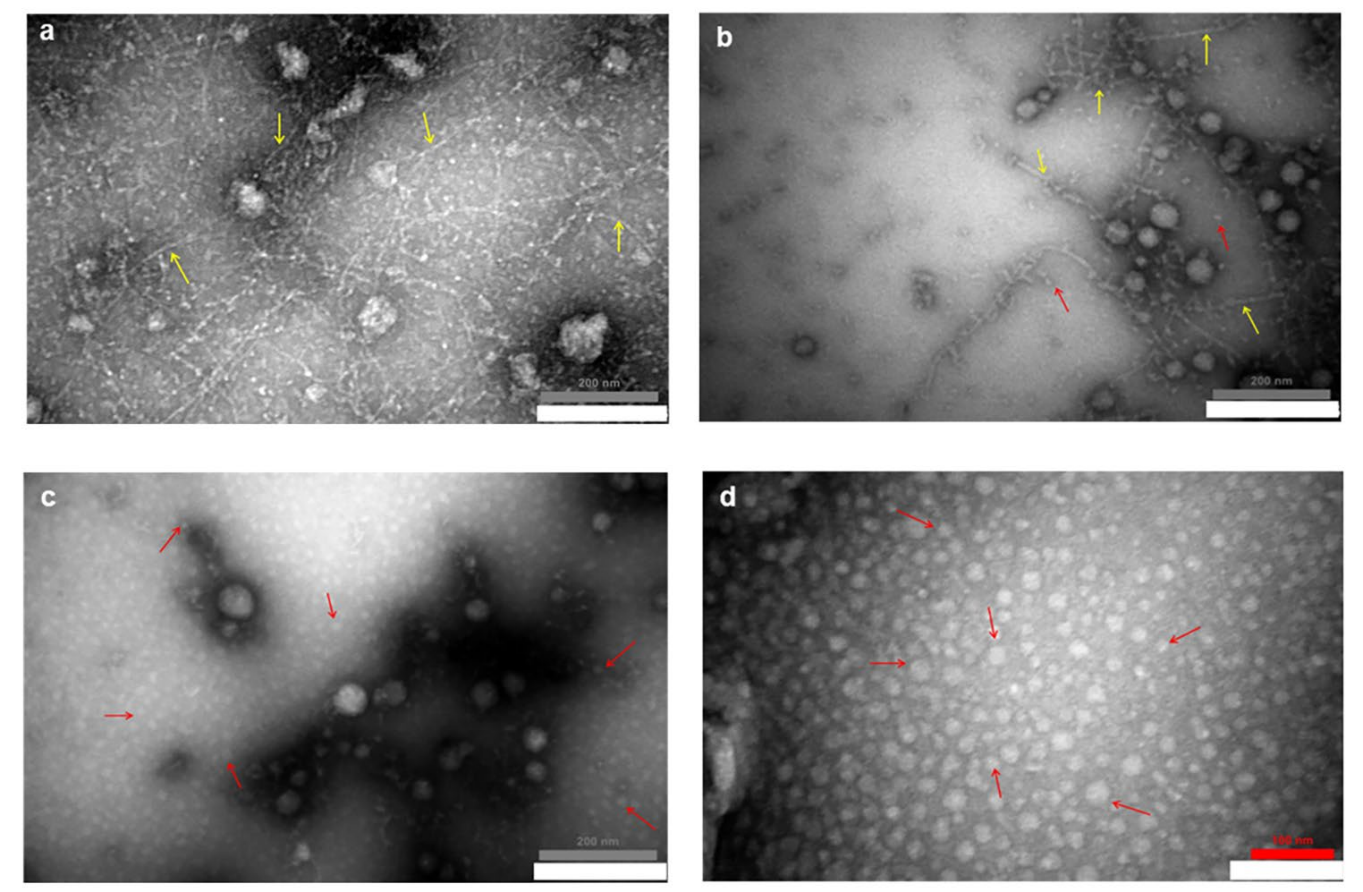
Transmission electron microscopy (TEM) images of different forms of Aβ_**1−42**_. **(a)** The non-ultrasonicated 100 µM (NUSH), **(b)** The ultrasonicated 100 µM Aβ_1−42_ (USH), **(c)** The ultrasonicated 25 µM Aβ_1−42_ concentration (USL), and **(d)** The oligomer conformer. TEM analysis showed that Aβ oligomeric species present more in the 25 µM concentration than 100 µM concentration of Aβ_1−42_. The yellow arrows show fibrils and red arrows show globular oligomers

TEM analysis was performed to obtain information about the shape and size of ultrasonicated Aβ_1−42_ aggregates. Figure 1a shows long fibrillar aggregates of NUSH, while Fig. 1d shows Aβ_1−42_ oligomers with spherical structures. Figure 1b and c show TEM images of USH and USL of Aβ_1−42_. Comparing these two figures indicate a marked decrease in fibrillar size and the effective fragmentation of amyloid fibrils to smaller species and oligomers at low concentrations of Aβ_1−42_ solution.

### **Western blot proProfiles Aβ**_**1−42**_ **pepPeptides difDifferentnditions**

**Fig. 2:**
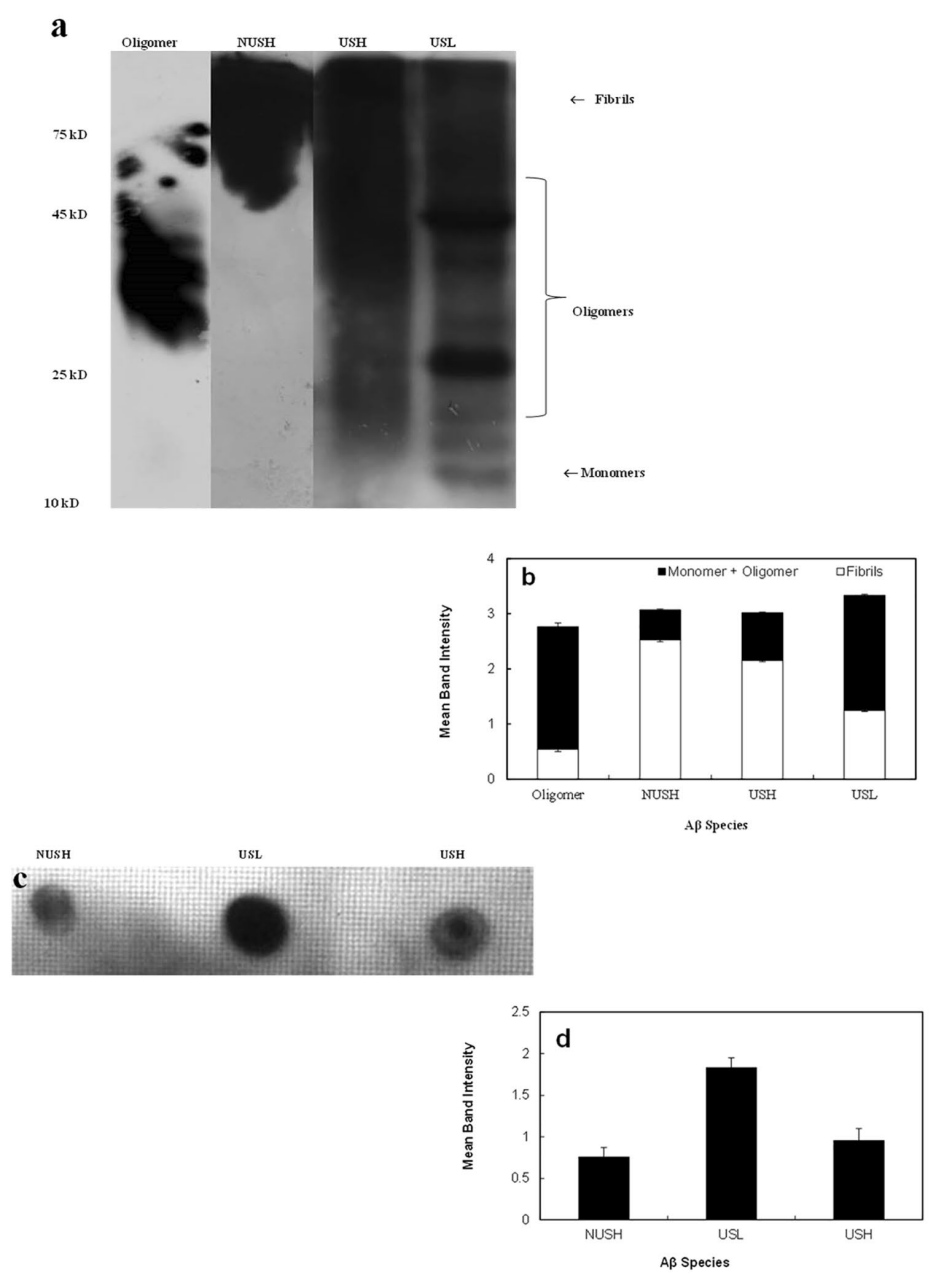
Western blot and Dot blot data of different forms of Aβ_**1−42**_. **(a)** Western blot analysis of different forms of Aβ_**1−42**_. Lanes 1 to 4 show oligomers, non-ultrasonicated 100 µM (NUSH), ultrasonicated 100 and 25 µM (USH and USL, respectively). Aβ_1−42_ disrupted due to ultrasonication to fragments with different sizes. **(b)** The related histogram of semi quantitative analysis of the bands in Western blot using *ImageJ* software. **(c)** Dot-blot analysis of the mentioned samples. **(d)** The histogram shows semi quantitative analysis of the dot blot spots of NUSH, USH and USL. The results are expressed as mean density of indicated bands ± SD based on three independent experiments

Figure 2a shows the Western blot bands. From left to right, it shows the oligomeric form of protein in lane 1, and the fibril with no ultrasonication in lane 2. Lanes 3 and 4 show the ultrasonicated high and low concentrations Aβ_1−42_ peptides, respectively. As can be seen, ultrasonication breaks proteins and smears appear. However, two 45 and 25 kDa bands at lower concentrations (USL) indicate oligomeric structures. Figure 2b is the histogram of Western Blot data, indicating the portion of monomer + oligomer vs. fibrils in each sample.

Figure 2c shows Dot blot spots. It indicates the smaller species content at low and high concentrations of Aβ_1−42_ peptides after ultrasonication. Figure 2d, the histogram of Dot blot data, indicates that the oligomeric form was considerably higher at USL (Aβ_1−42_25 µM, after ultrasonication) than in others.

### ThT Fluorescence Intensity and ANS Binding Assay

**Fig. 3:**
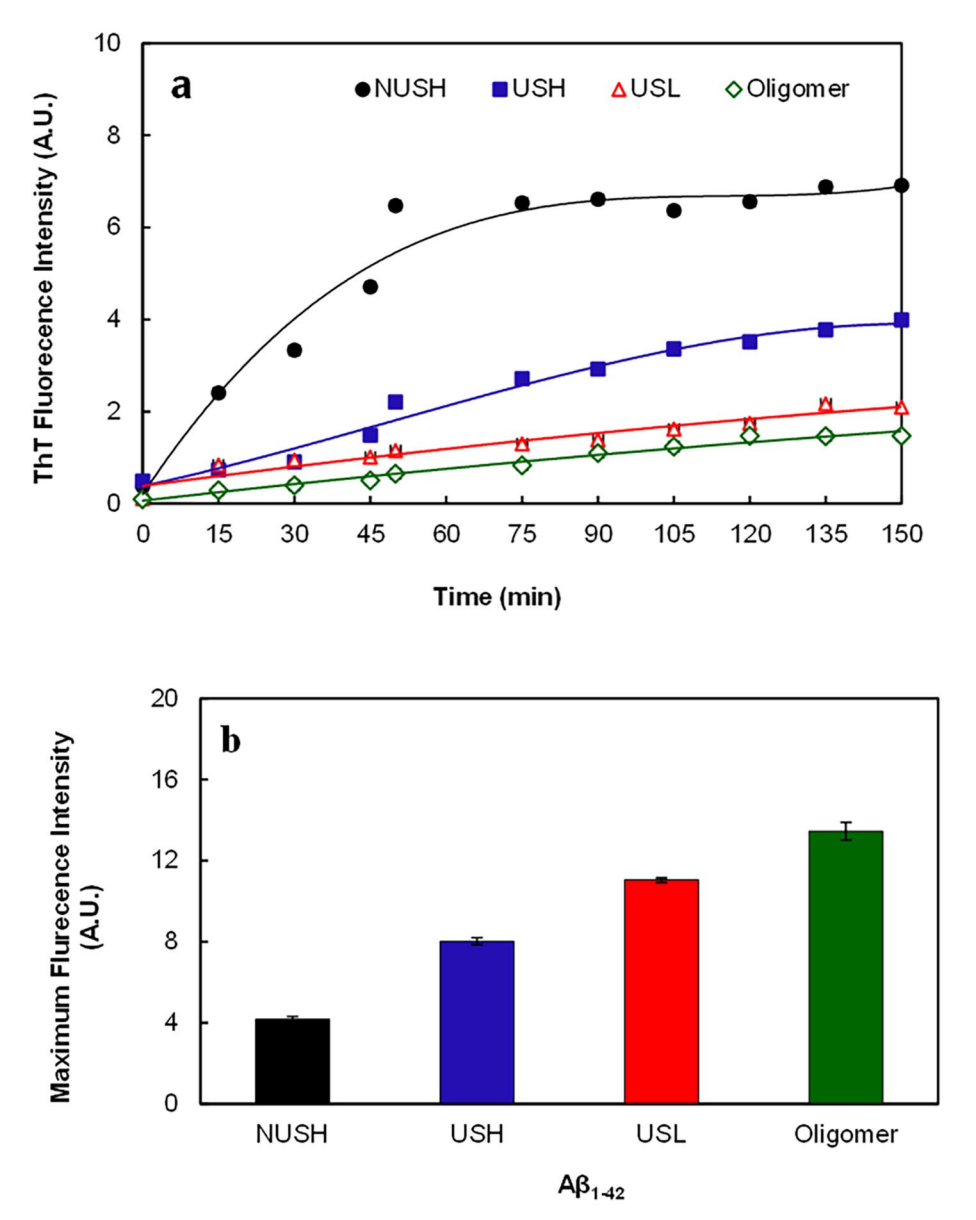
ThT and ANS fluorescence intensity of Aβ_1−42_ at different conditions. (a) Fluorescence intensity of the ThT-treated Aβ_1−42_ peptide at different conditions (ultrasonicated 25 and 100 µM as USL and USH, respectively; non-ultrasonicated 100 µM as NUSH and oligomer) and different time intervals; where λ_ex_ and λ_em_ was 450 and 482 nm, respectively. **(b)** ANS fluorescence intensity after 150 min incubation with different forms of Aβ_1−42_, where λ_ex_ and λ_em_ was 380 and 500 nm, respectively. All results shown are representative of at least three independent experiments, and the results are shown as means ± SD.

The interaction of Aβ_1−42_ peptides with ThT, a dye reactive with β-sheet-rich conformers, and ANS, a hydrophobic dye, were studied. Figure 3a shows the enhancement of ThT fluorescence emission following its incubation with different forms of Aβ_1−42_ peptides. NUSH showed higher fluorescence intensity after interaction with ThT, which was increased up to 3 h. However, the ultrasonicated forms showed lower fluorescence intensity changes. Figure 3a shows that the lowest ThT fluorescence intensity belongs to oligomer and USL of Aβ_1−42_, indicating the lowest fibrillar form.

Figure 3b shows the results of the ANS binding assay. The highest ANS fluorescence intensity was observed in the solutions containing oligomers and USL of Aβ after 150 min incubation. In contrast, the lowest fluorescence intensity was observed in the NUSH solution. It indicates the formation of Aβ_1−42_ species with higher surface hydrophobicity, such as those observed in Aβ oligomers in the USL. These results suggest that ultrasonication shifts the equilibrium toward Aβ_1−42_ fibrils depolymerization, monomer, and oligomer formation. Furthermore, these changes were more at lower Aβ_1−42_ concentrations.

#### Secondary Structure of Different Forms of Aβ_1−42_

**Fig. 4:**
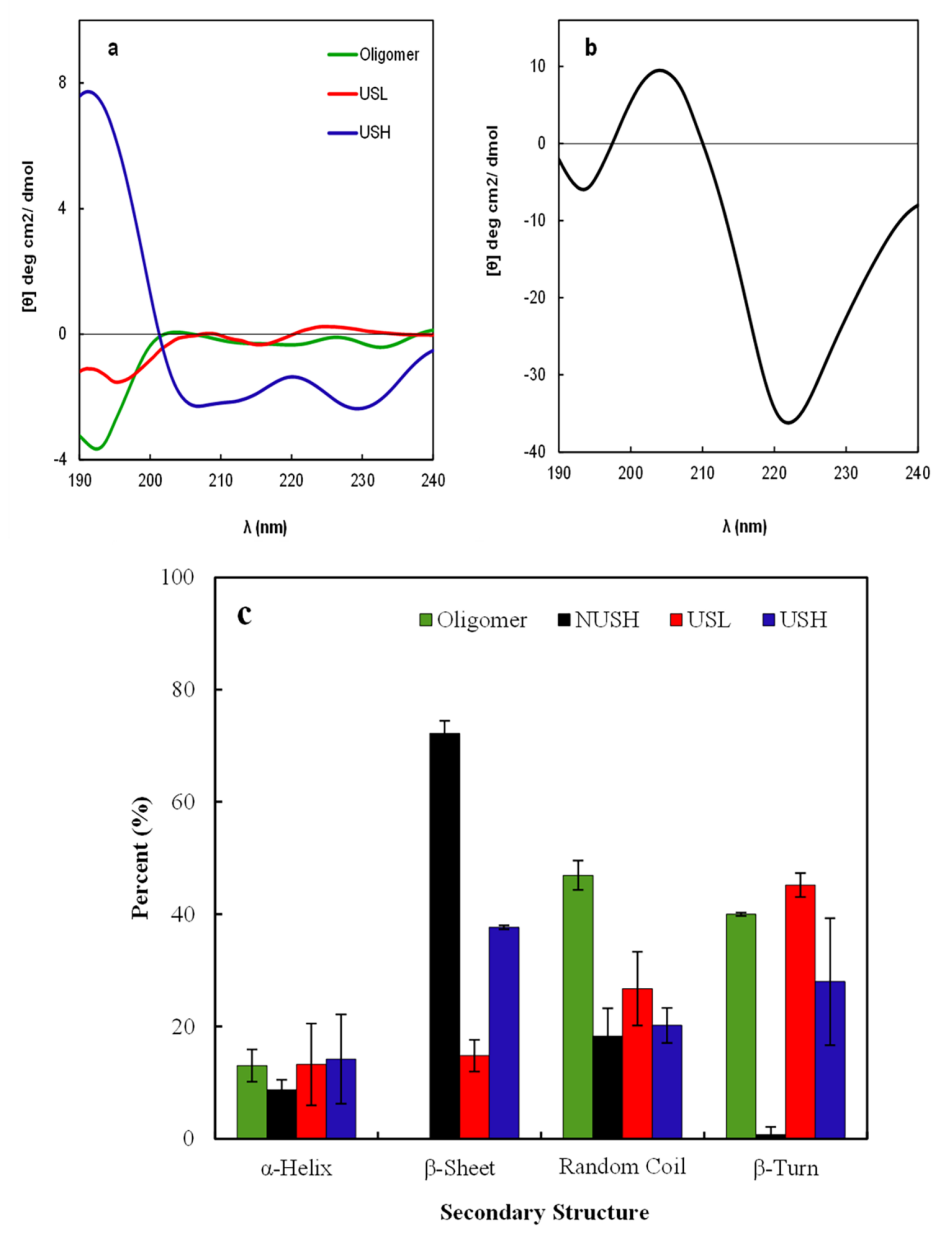
Characterization of the Aβ_1−42_ secondary structure after ultrasonication. **(a)** The Secondary structures of oligomers, USH, and USL (100 and 25 µM of ultrasonicated Aβ_1−42_, respectively); **(b)** The CD plot of NUSH **(**non-ultrasonicated, 100 µM) Aβ_1−42_. It shows a characteristic negative peak around 220 nm of β-sheet structure for NUSH. After ultrasonication the Secondary structures for the peptides in solution were changed. **(c)** shows the percentages of the Secondary structures at different situations as estimated by the software

Figure 4a and c indicate oligomers’ higher percentage of β-turn and random coil (40.0% and 46.9%, respectively). The NUSH solution showed a β-sheet-rich structure for fibrils (Fig. 4b and c), while after ultrasonication, at higher concentration, the percentages of β-sheet decreased (from ~ 72.2% to ~ 37.6%) at the expense of β-turn (from 0.8 to 28.0%) and random coil (from 18.3 to 20.2%) conformations. At lower concentrations, the β-turn was (45.2%) higher than in other structures (Fig. 4c).

### Cell Toxicity Assay of Aβ_1−42_ at Different Conditions

**Fig. 5:**
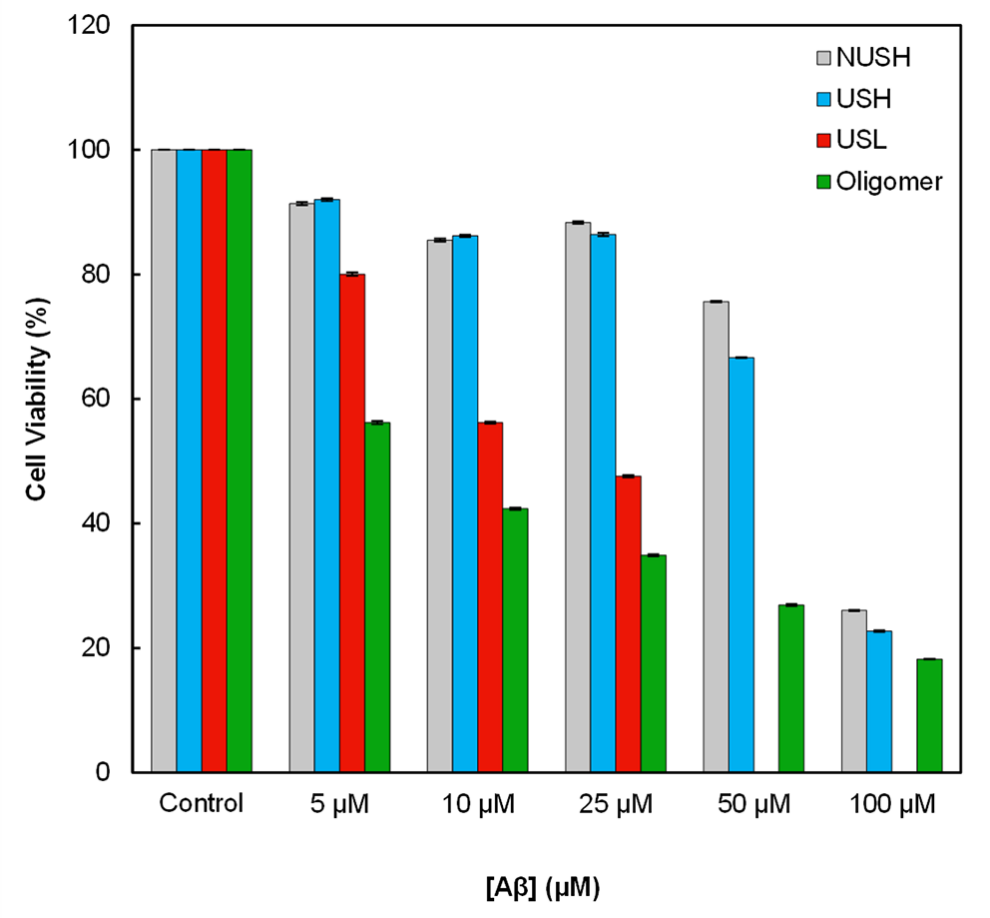
The effect of different forms of Aβ_1−42_ on PC12 cell viability. Toxicity of different forms of Aβ_1−42_ NUSH (100 µM) and USH (ultrasonicated 100 µM) and USH (ultrasonicated 25µM), as well as Aβ_1−42_ oligomers was measured by MTT assay after 24 h. In this experiment, different concentrations of the ultrasonicated and non-ultrasonicated solutions were used. The order of toxicity of different structure was oligomers > USL > USL > NUSH. The graph represents the mean ± SD from triplicate wells

The toxicity of different forms of Aβ peptide against PC12 cells was investigated using MTT assay and expressed as percentages of cell viability. The results in Fig. 5 indicated more toxicity of ultrasonicated Aβ_1−42_ peptide (USL) than non-ultrasonicated one (NUSH) against these cells. The IC50 of oligomers was about 5 µM, and the IC50 of the USL was 10 µM, which is the highest cytotoxicity compared to other forms.

### Uptake of Aβ_1−42_ into the PC12 Cells

**Fig. 6:**
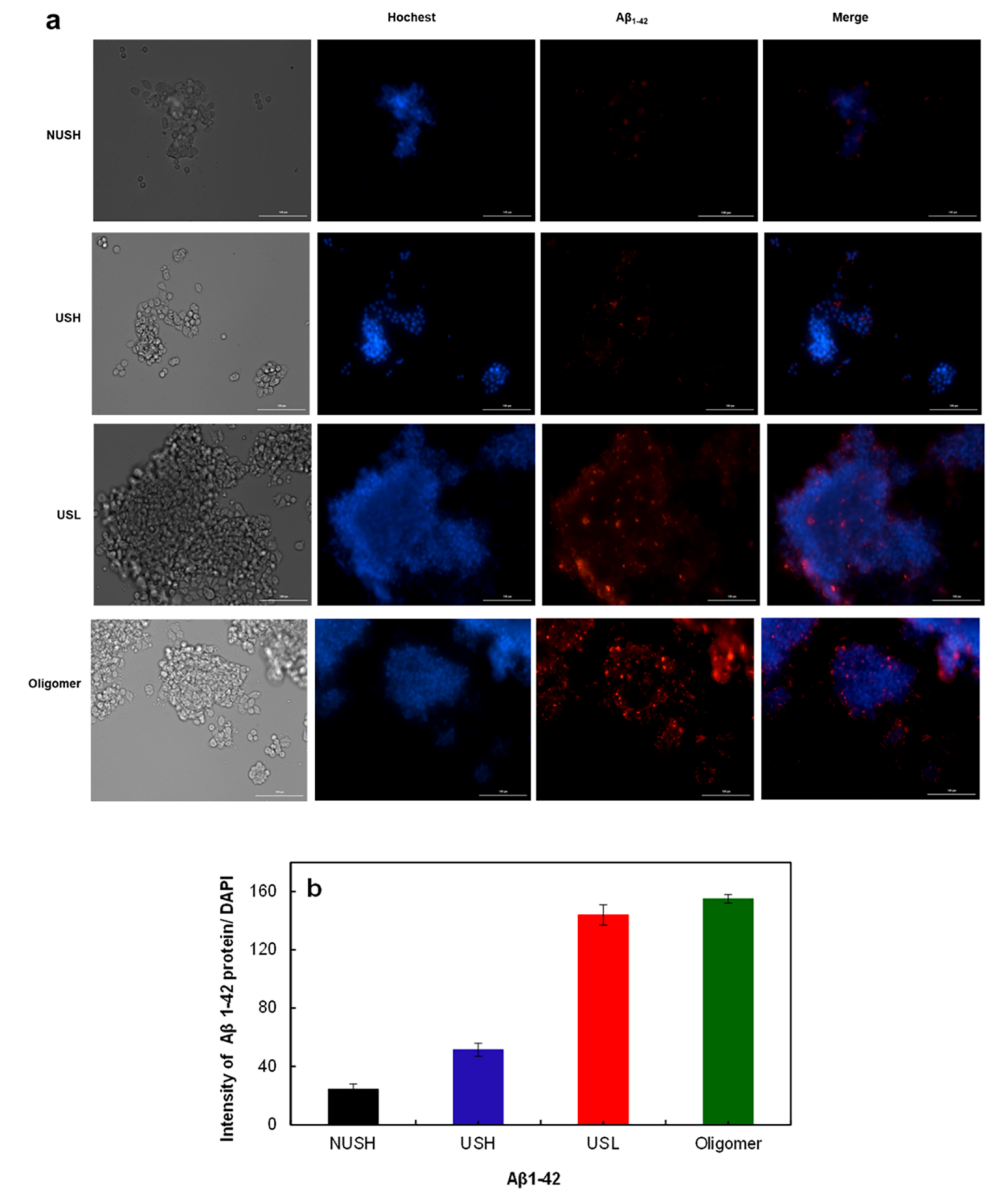
PC12 Cell uptake of Aβ_1−42_. **(a)** PC12 cells that were cultured in the presence USL and USH (ultrasonicated 25 and 100 µM), NUSH (non-ultrasonicated 100 µM) of Aβ_1−42_ and oligomers for 24 h. Then, they were exposed to primary Aβ_1−42_ antibody, Alexa Fluor 594 and Hoechst 33,258 and images were prepared with fluorescent microscopy. The Aβ_1−42_ uptakes observed in USL (ultrasonicated low concentration (25 µM)) more than USH (higher concentration (100 µM)) before and after ultrasonication. **(b)** The histogram shows the ratio of red color intensity of Aβ_1−42_ to the cell nuclear number, in PC12 cells. All results shown are representative of at least three independent experiments, and the results are shown as means ± SD.

The Aβ uptake by PC12 cells was studied by incubating the cells with a primary antibody against the Aβ_1–42_ peptides. Then, cells were stained with a secondary antibody against Aβ_1–42_ and Hoechst 33,258 for the nucleus. Figure 6a and b show the maximum and significant (p = 0.000) cellular uptake in cells exposed to the ultrasonicated-25 µM (USL) and AβOs.

## Discussion

In the present study, stable Aβ_1−42_ fibrils (erroneously formed at 220 µM in a phosphate buffer containing NaCl incubated at 37 °C) were disaggregated through ultrasonication and then, refolded to oligomers. To optimize the method, we first tried to form the Aβ fibrils at high and low concentrations (100 and 25 µM) and oligomers by the standard methods of Stine et al. [25]. Then, we evaluated some properties of Aβ_1−42_ before and after ultrasonication. Our findings show that ultrasonicated-25 µM of Aβ_1−42_ was more toxic against PC12 cells than 100 µM before or after ultrasonication. The Western blot data showed Aβ_1−42_ fibrillation, mostly at 100 µM, before ultrasonication. Due to ultrasonication, the fibrils broke through into smaller species, resulting in oligomer formation. However, a low concentration of Aβ_1–42_ adopts a predominantly smaller size aggregate and oligomeric structure. The changes in the fluorescence intensities of different forms of Aβ_1−42_ in the presence of ThT and ANS were also other affirmative reasons for this finding. Dot blot analysis also indicated the presence of higher amounts of monomer and oligomer species after ultrasonication of 25 µM preparations than in the 100 µM preparations. We also showed the differences in the Aβ_1−42_ structures at high and low concentrations using TEM images. Given the toxicity assay, we showed that USL was more toxic than other forms for PC12 cells due to their uptake by the cells, confirming their oligomeric structure.

More than 80 functional or pathologic amyloid fibril proteins have been characterized [27]. However, forming Aβ_1−42_ fibers may happen in two different in vitro situations. Firstly, a controlled situation using a certain peptide concentration in the presence of heparin, dithiothreitol (DTT), and temperature [28]. These types of aggregates may be disaggregated in the presence of some peptides or small molecules as inhibitors [29, 30].

The second is an uncontrolled random situation due to inappropriate very high concentration, buffer, temperature, pH, some ions like Cu^2+^, agitation, and so on [31]. These types of fibrillization are not inhibited readily and cause severe problems in experimental situations and use Aβ peptide in the experiments.

In the present study, we tried to disaggregate the fibrils of the second category, which are very stable, using ultrasound and a suitable buffer, and then convert the monomers into oligomers. For this purpose, we used various methods to characterize the aggregates.

The ThT-binding assay is a method of choice for detecting the presence of amyloid fibrils [32–34]. It showed a higher fluorescence intensity of NUSH, indicating more amyloid fibril formation. In contrast, the USL displays the lowest ThT fluorescence intensity, indicating the lowest fibrillar content. These data are consistent with the data obtained by TEM analysis. TEM examination of incubated NUSH also showed a tremendous amount of amyloid fibril content, while the lowest amount was observed at 25 µM solution after ultrasonication.

In addition, the highest ANS binding to the USL is another reason for Aβ oligomer formation. ANS is highly sensitive to the polarity of peptides and proteins. The fluorescence intensity increases upon interaction with the exposed hydrophobic regions in native or partially unfolded proteins [35]. Ultrasonic disruption of the fibrils increased to the extent of solvent exposure of hydrophobic Aβ_1−42_ fragments and led to evaluate the hydrophobicity of Aβ using the ANS-binding assay. Aβ_1−42_ oligomers are more hydrophobic [18] and, thus, bind more ANS. While hydrophobicity of both NUSH and USH were lower than USL.

Aβ_1−42_ fibrils are composed of two in-register inter-strand parallel β-sheets connected by a bend between residues 25 and 30 [5, 36, 37]. Residues 10–16 are part of the first β-sheet in fibrils [11, 36, 37] and disordered in the pre-globulomer [38]. Two hydrophobic peptide segments within Aβ_1−42_, residues 16–22 and 30–42, are solvent-exposed in the toxic Aβ_1−42_ oligomers [18]. We also evaluated the secondary structure of the peptides by CD spectroscopy. The spectrum of NUSH would possess a sharp negative peak at about 220 nm, indicating that the peptide adopts mainly (~ 60%) the β-sheet conformation. In contrast, the spectra of other peptides show different characteristic peaks, indicating the decreased β-sheet percentage at the expense of β-Turn and random coil formation.

We also investigated the effect of ultrasonication on Aβ_1−42_ fragmentation. According to the Western blot analysis, ultrasonication of 25 µM of Aβ_1−42_ induced a significant increase in the densities of the bands around 20–60 kDa. This pattern is compatible with the formation of Aβ_1−42_ monomers (~ 10 kDa), oligomers (~ 25 kDa), and fibrils (~ 75 kDa) [39–41]. These results are in excellent agreement with those obtained from Dot blot in which the antibody recognized oligomers in USL more than those in USH solutions of Aβ_1−42_. Furthermore, these data are compatible with the model that demonstrated two distinct pathways for the amyloid Aβ_1−42_ soluble (oligomer) and insoluble (fibril) peptides [6, 42]. Similar studies have been performed on 25 µM [43] and 8 µM [44] of the Aβ_1−40_ peptide, illustrating the role of environmental factors in the peptide structure.

A switch in mechanism with concentration has also been observed with Aβ_1−42_. In a narrow concentration range (20 − 25 µM), spherical Aβ_1−42_ oligomers formed which were positive for the oligomer-specific A11 antibody and showed a high capacity to disrupt lipid bilayers [18]. By contrast, at higher concentrations, a different type of oligomer was formed that did not react with the A11 antibody that did not disrupt lipid bilayers, despite having a similar size and secondary structure at low concentrations (< 20 µM) [18]. Studies such as these show the importance of recognizing the influence of concentration-dependent mechanistic changes and the need for accurate concentration measurements before experiments start[17].

Previous studies have shown that the Aβ_1−42_ amyloid fibrils in the membrane consist of two intermolecular β-sheets: β1, which is in the non-transmembrane (NTM) region (residues 17 − 28), and β2, is in the transmembrane (TM) region (residues 29 − 42) [5]. Under ultrasonication, a bubble was usually created around hydrophobic residues in the TM region after pressure becomes negative. The assembly of hydrophobic residues in the TM region acts as a nucleus for bubble formation, and the bubble breaks down the fibrils. The amyloid fibrils keep their β-sheet structure even in the bubble. When the pressure becomes positive, the bubbles shrink and collapse, water molecules crash against the hydrophilic residues in the NTM region, and then amyloids disrupt. The deformation and disruption of the β-sheet structure at the NTM region are possible because of the presence of Glu22 and Asp23 with negative electric charges. The repulsion between these residues is shielded in water but not in the bubble, which is the main reason for β-sheet structure destruction in the NTM region in the bubble. In the case of short amyloid fibrils like trimer, hexamer, and dodecamer, the Aβ peptides do not have enough hydrophobic residues to create a bubble. Thus, amyloid fibrils do not disrupt. Therefore, more time of negative pressure is required for bubble formation [22]. It appears that following ultrasonication, the potential for formation of antiparallel β-sheet at low Aβ_1−42_ concentrations is greater than its high concentrations. It may be due to the repulsion between the negatively charged amino acids, Glu22 and Asp23, and the critical concentration of micelle-like formation. It has shown that after ultrasonication of both low and high concentrations, Aβ_1−42_ fibrils break down into monomers and smaller species of aggregates. However, the kinetic, thermodynamic stability, and energy landscape differences lead to possible conformations.

## Conclusions

We examined the effect of the ultrasonic application on low and high concentrations of Aβ_1−42_ peptide to study the simultaneous effects of the concentration and ultrasonication on Aβ_1−42_ fibril disruption. The results indicated that due to ultrasonication of low concentration of Aβ_1−42_, long amyloid fibrils disrupted and fragmented to smaller species of aggregates, including 2–12 mers. Furthermore, these small spherical Aβ_1−42_ aggregates were more toxic against PC12 cells than the fibrillar aggregates at higher concentrations of Aβ_1−42_. In addition, their characteristics were very similar to Aβ oligomers. The ThT and CD studies indicated the following order for β-sheet content in the Aβ_1−42_ peptides: NUSH > USH > USL > Oligomers.
